# Facts and Cases in Obstetric Medicine, with Observations on Some of the Most Important Diseases of Females

**Published:** 1837-01-01

**Authors:** 


Facts and Cases in Obstetric Medicine, with Observations on some of the most important Diseases incidental to Females. By *J. T. Ingleby*, Member of the Royal College of Surgeons in London, Senior Surgeon to the General Dispensary; Surgeon to the Magdalen Asylum; and Lecturer on Midwifery at the Royal School of Medicine, Birmingham. Octavo, pp. 296. London. Longman and Co.

Whether we view its splendid museum, the largest out of the metropolis, and chiefly collated by its illustrious founder, Mr. Sands Cox—the number and proficiency of its students, amounting to nearly one hundred—the zeal and talents of its teachers—and the extent and variety of its instructions, which have recently included the study of mathematics; the school of medicine at Birmingham has acquired a celebrity, not inferior to that of any similar institution. While the elements of professional knowledge are thus placed within the reach of every disciple, it is a subject of serious regret that the wards of one of the largest and best conducted hospitals in the provinces are open only to a limited portion of the students thirsting for practical information, and lamenting the absence of clinical tuition. The number of beds contained in this establishment, which exceeds that of some of the recognized hospitals in London, and the high repute which its medical and surgical officers have acquired, are surely sufficient to satisfy the *purest* and most fastidious practitioners that the principles and practice of medicine and surgery may be as scientifically and successfully taught within its walls as at any college in the kingdom. We are aware that these sentiments differ from the opinions of the distinguished surgeons of this excellent charity, expressed some time ago. Nevertheless, from our own knowledge of their experience and operative skill, we are convinced that the students at Birmingham would gladly avail themselves of access to the hospital under reasonable restrictions, and as fully appreciate the liberality, which may afford them clinical information, as they already do the free admission to the operating theatre, which we must do the surgeons the justice to say they have generously permitted them to enjoy. Were this valuable institution rendered subservient to the dissemination of science as well as philanthropy and its portals thrown open to the profession, not only would the younger members reap a luxuriant harvest, but the medical and surgical officers might render important services to society by arranging and communicating the rich stores of information which such an establishment cannot fail to afford them. To the march of time however, which, as Lord Bacon observed, is the great reformer, we must leave this matter, and proceed to the immediate object before us.

Mr. Ingleby's *labours* in obstetric science have already been sufficiently *prolific* to justify us in considering him zealous and diligent in his legitimate department. His work on uterine hæmorrhage, noticed by us in the 33d number of the new series of our Review, has already gone through one edition, and we believe the industrious author is revising his useful performance for re-publication. The favourable opinion we gave of that, his first effort, has been confirmed by the sale of every copy of his book.

The present work is divided into seven sections :—1. " *On Puerperal Convulsions ;* 2. *On Malposition of the Uterus, Ovaria, Bladder, and Urethra, both in the impregnated and unimpregnated state, in connection* (connexion) *with Retention of Urine ;* 3. *On Obstructions in the soft parts to the Progress of Labour ;* 4. *On the Induction of Premature Labour in Cases of Organic Disease ;* 5. *On Laceration of the Uterus and Vagina ;* 6. *On Inversion of the Uterus ;* 7. *On the Signs and Symptoms of Pregnancy, their obscure and deceptive Characters, their Complication with Disease, and the Signs which denote the Extinction of Life in the Fœtus ; with an Appendix to Section* 6."

As the first three and a portion of the sixth and seventh sections are already in the hands of the profession, we shall briefly notice their contents, and pass on to the more original portions of the work.

1. After alluding to the different forms of puerperal convulsions, the author observes, by way of distinction from hysteria, that the latter is accompanied

"Neither with stertor or (nor) coma, and rarely, if ever, by convulsion of the muscles of the face." P. 3.

Our own experience corresponds with that of Sydenham, who says—

"This disease is not more remarkable for its frequency than for the numerous forms under which it appears, resembling most of the distempers wherewith mankind are afflicted.—*Swan's Translation,* 1749, *p.* 370.

Dr. Conolly, in his article on Hysteria, in the Dictionary of Practical Medicine, remarks, that the fit consists sometimes of a temporary palsy of some of the voluntary muscles, and—

"Sometimes of all the voluntary muscles of the body, and the patient falls into a state of coma; is insensible, &c."

"These cases somewhat resemble apoplexy, and cases are on record in which they have gone on to it."—*Vol.* 2, *p.* 560.

Our object for noticing this Proteiform character of hysteria is to warn our junior readers from relying too implicitly, in forming their diagnosis, on isolated symptoms; as all experienced practitioners must admit that the line, which separates this disease from others, which it resembles, is too delicate for superficial observation.

With respect to the causes of puerperal convulsions, the author states that they may be referred to—

"Either an excited or turgid state of the vessels of the brain (often promoted by improper diet and a neglected state of the bowels during gestation) or by (to) loss of blood, as after a dangerous hæmorrhage. There is also a third state subordinate to those just mentioned, and which seems more immediately dependent upon excessive sensibility of the uterine fibres, since it generally happens under an irregular and highly painful action of the uterus during its dilatation." 9.

The anasarcous swellings are properly considered by Mr. Ingleby as concomitants, and owing to one common cause, namely, an embarrassed circulation.

After detailing the various symptoms of convulsions the author observes—

"I am acquainted with several cases of puerperal convulsions, which were succeeded by puerperal mania: the transition may probably be the result of the large bleedings, which were necessary to subdue the primary disease." 17.

We should be inclined to attribute the madness in these instances to an inflammatory state of the vessels of the brain or its investing membranes: the large bleedings having removed the congestion in the cerebral circulation, and unfolded the mental derangement.

From the pathology and prognosis of puerperal convulsions we pass on to the treatment of the disease. In the sthenic species there are two indications to be attended to; the first to allay vascular turgescence and excitement; the second to diminish the size of the uterus by the evacuation of a part or the whole of its contents. The author is an advocate for early and active depletion, and in a plethoric condition of the system there can be no doubt of its propriety and great importance. Venesection at the arm or arteriotomy at the temple may be employed according to circumstances. After the fit has subsided, we must lose no time in the use of active purgatives. On this subject much useful information is given, for which and for other minutiæ in the treatment we must refer to the work itself.

Before the seventh month of pregnancy, the artificial evacuation of the uterus is improper under any circumstances. The membranes may be ruptured, and the liquor amnii discharged at a much earlier period. It is however questionable whether after the seventh month this operation should be performed, unless the state of the os uteri should render immediate delivery by turning readily practicable: as such a contraction of the uterus may succeed, as might render the subsequent introduction of the hand difficult or dangerous; or the uterine action, on which the ultimate benefit of the proceeding may depend, may not supervene at a seasonable period. Hence, as our author observes, convulsions preceding labour are highly dangerous and, according to Dr. Hall,

"In every case which terminated unfavourably, there was no dilatation of the os uteri, and consequently no labour." 32.

When convulsions occur after labour has commenced, and they appear to be of an hysterical nature, when the patient is sensible between the fits, and the pains are regular, the author remarks that the case may be left to Nature. On the contrary, when the disease is urgent, and the os uteri in a soft and yielding state, artificial delivery should be immediately accomplished. On the practice of dividing the rigid and undeveloped cervix uteri, adopted by Dubois, Mr. Ingleby makes some sensible remarks, and concludes by recommending the treatment adopted by Dr. Joseph Clarke in such cases, viz. to trust to the efforts of Nature, assisted by medical means, unless the life of the patient is endangered, and then to promote delivery in the speediest and safest manner.

"Convulsions," the author states, "arising about the second day after delivery, are usually connected with the secretion of milk, and demand active depletion; but subsequently to this period the attack will probably be connected with irritative fever, the result of decomposed portions of disrupted placenta, of which I have seen several instances.

Phegmatia dolens is a frequent sequel of this species of convulsion." 41.

In referring this irritative fever and phlegmatia dolens exclusively to decomposed portions of disrupted placenta, without the intervention of any secondary cause, we beg leave to differ from the author; as our experience and that of others have proved of late that these diseases are the result of inflammation and purulent deposit in the veins of the uterus, or the neighbouring venous tubes; in short, they constitute what is called uterine phlebitis, and may often be successfully combated by leeches applied to the vicinity of the inflammation, and by an early and judicious use of calomel and opium. In such cases, as the author justly states, the depleting system is rarely admissible. His directions for the management of patients, labouring under this variety of irritative or typhoid fever, are full and peculiarly applicable.

Those puerperal convulsions resulting from hæmorrhage are most successfully treated by opium. Here secale cornutum, ammonia, aromatic confection, and other stimulants and cordials, have been found by the author appropriate remedies. Various collateral means are mentioned; and the

Essay concludes with numerous cases, which our limits will only allow us to recommend for perusal.

2. In speaking of retroversion of the uterus, Mr. Ingleby advocates the opinion of Dr. Merriman, that this organ may undergo a partial retroversion during the whole period of pregnancy; and he also extols the frequent use of the catheter, recommended by the latter in these cases. In obstinate retroversions, the author advises an attempt to be made to replace the dislocated organ, and details the usual plan of proceeding. The absolute necessity for this manual interference must be very rare, except where the pelvis is distorted, as we have never had occasion to adopt it, although engaged in an extensive private and consulting practice. In one instance, the catheter was introduced regularly from the third to the fifth month, when the ascent of the uterus gradually and completely followed. The use of this instrument has been found in some cases impossible, and the patients have died from ruptured bladder.

A peculiar form of incomplete retroversion sometimes occurs, in which the os uteri rests above the symphysis pubis. The bladder in this case is liable to undergo enormous distention. Dr. Weir of Glasgow succeeded in a case of his kind in restoring the natural position of the displaced organ, by pushing upwards the fundus, and pulling downwards the os uteri.

A distorted direction of the urethra will occasion difficulty to the surgeon, and require a corresponding curve in the catheter.

When the disease is occasioned by morbid enlargement of the body or fundus of the uterus, this organ must not be permitted to continue in a state of retroversion.

Complicated retroversions and flexions of the uterus next engage the author's attention; and after making some useful remarks on malposition of the bladder, illustrated by two interesting cases, he terminates this part of the volume with some practical observations on malposition of the urethra.

3. Tumors in the labia pudendi, occasioned by extravasation of blood within the cellular membrane, and obstructing the progress of labours, may be safely relieved by punctures, effected by means of a very fine curved needle.

Contractions of the vaginal orifice and canal, impeding the process of parturition, will generally give way to the natural efforts. When incisions are required, the author agrees with Dr. Rigby in recommending them to be made towards the termination of labour, and during one of the pains.

After the incised parts have been healed, it will in such cases be needful to prevent a recurrence of the contraction by the regular employment of bougies.

The fluctuating ovarian tumors, impeding labours, may be punctured, when situated in the recto-vaginal septum. Tumors, not admitting relief by this proceeding, may be extirpated, or require the use of the perforator. An ovarian cyst may present in other situations. It has been said to be found at the anterior part of the pelvis, and in a lady, who had been under the care of two eminent lecturers on midwifery in London, and afterwards consulted Mr. J. M. Coley of Bridgnorth, a tumor of this description was discovered by him presenting at the side of the pelvis.

Fibrous tumors in the fundus of the uterus present no impediment parturition, and may co-exist with pregnancy, when not obstructing the opening in the fallopian tube. When they extend towards the cervix uteri, they present insuperable obstacles to delivery by the natural process. As many of these uterine tumors are found to contain a fluid, the author recommends the operation of puncturing, and submits for consideration the propriety of introducing a needle or fine trochar.

After treating of polypus in utero, and the excision of tumors from the vagina and uterus, the author terminates his remarks with an important case, in which a fibrous tumor was successfully removed, communicated by one of his pupils.

4. In the section on the induction of premature labour in cases of organic disease, the author alludes to the novel application of this measure by Dr. Ashwell, published in Guy's Hospital Reports. One of the diseases, for which this operation is recommended, is the fleshy or fibrous tubercle.

This fibrous tumor is met with of various size, from that of a pea to that of a cricket-ball, or still larger. Its shape is generally globular, and its structure semi-cartilaginous, disposed in a radiated form. Occasionally it is granular in its appearance, and resembles a mass of minute tumors, having separate envelopes. In the firmest tumors the interior is sometimes found in a soft or liquid state. In other instances, as the disease advances, its density increases, until it acquires the consistency and character of cartilage without any vessels containing red blood. Calcareous depositions are occasionally observed in the morbid structure, which by degrees acquire all the properties of carbonate and phospate of lime, varying in consistence from that of pumice-stone to that of marble.

"The presence of the fleshy tubercle in the unimpregnated uterus is rarely attended with immediate danger to life. The distress will be either of a neuralgic or subacute kind. It is the combination of pregnancy with this disease of the uterus, which so materially affects the welfare of the patient." "The tumours (according to Dr. Ashwell) soften during the latter months, the increased vascular supply leads to increased inflammation, unhealthy and imperfect suppuration is established in them, and death occurs soon after parturition." "With the view of preventing or removing inflammation, and averting the pressure and contusion, which the gravid uterus is supposed to make upon the diseased growth, Dr. Ashwell strongly recommends the induction of premature labour." 157.

The author doubts the existence of this disorganization so frequently as believed by Dr. Ashwell, and adduces two cases in support of his assertion. He appears to be of opinion too, that death following parturition is attributable to peritoneal inflammation, as was found to be the case in these instances, and as may be inferred from the following passage.

"When fibrous tumours exist in the gravid uterus, the structure surrounding them is more vascular than when they exist in that organ in the unimpregnated state. It would thus appear that the tumours will be more predisposed to put on inflammatory action in the former than in the latter state of the uterus. Granting then that the tumour becomes inflamed during gestation, is it warrantable to have recourse to an operation so responsible as the one in question? In all acute diseases is not the danger known to be greatly increased during a state of pregnancy, the premature expulsion of the ovum being a common result of the prevailing excitement? The propriety of bringing on labour, whilst a tumour is in a state of active inflammation, may very justly be questioned; for the contraction of the uterus, necessary to accomplish the separation of the membranes, and the expulsion of the fœtus, would most probably increase the irritation in the tumour, and thus exasperate the inflammation." 158.

Now it does not appear to us that inflammation is more likely to be brought on by the uterine action consequent on a careful evacuation of the liquor amnii, than by that attendant on labour at the full period of utero-gestation, which has spontaneously supervened. Inflammation is not, however, the only consequence to be dreaded in these cases from labour artificially or naturally excited: as hæmorrhage arising from an inability in the uterus to contract, is an affair of equal, if not far greater importance. From this cause alone death has been found to take place in a short time after the birth of the child, as was witnessed by Chaussier, and adverted to by Dr. Lee in a paper on fibro-calcareous tumors, published in the 19th Vol. of the Transactions of the Royal Medical and Chirurgical Society. The favourable termination of the two first cases suggesting to Dr. Ashwell the proceeding he recommends, in which premature labour was unintentionally induced by the requisite mode of examination, appears to us to justify the attempt. When, therefore, the co-existence of a fibrous tumor and pregnancy can be satisfactorily ascertained, and the situation of the disease is favourable for the process, we are of opinion that the premature rupture of the membranes might be followed by the happiest results; and, should the bulk and situation of the tumor totally preclude the expulsion of a living fœtus at the seventh month, that the operation may be performed at the fourth or fifth, by the introduction of a small male silver-catheter, at which early period, should contraction of the uterine parietes be found impossible, a fatal hæmorrhage would not be so likely to occur as at a more advanced stage of gestation; and it may be restrained by the application of the plug, which the author, in his work on uterine hæmorrhage, has so much and so justly extolled. The pain and distress resulting from the unalterable confinement of the morbid uterus within the pelvis, might be another motive for the adoption of the modification we have proposed of the treatment recommended by Dr. Ashwell. At the same time we concur with Mr. Ingleby in reprobating the indiscriminate adoption of the practice, as in the hands of inexperienced practitioners it might bring an useful operation into unmerited disrepute.

Nature sometimes in these cases effects that, which by art we have been in vain endeavouring to accomplish. A diminution, often sudden, begins in the tumefied mass, which may subside within the natural boundaries of the uterus. Of this fact the author seems to be fully aware, and in doubtful cases we think it should be taken into consideration as a possible event. We are not aware, however, that such a result has hitherto been noticed in the impregnated uterus.

The pain attending the development of fibrous tumors is various, probably depending in some measure on the direction they may take. Originating in the fibrous structure of the uterus, they sometimes extend to its serous and sometimes its mucous coat. In the former case the symptoms of acute or chronic peritonitis will present themselves, in the latter increased discharge with or without blood will attend, and the tumor, delivered into the vagina through the gradually dilated orifice of the uterus, may assume the character of polypus, and be safely removed by operation. When acute inflammation from any case attacks the peritoneal coat of the uterus, adhesions between its free surface and that of the abdominal peritoneum and the omentum are apt to follow, and to simulate by their density and induration fibrous and malignant tumors. An instance of this kind was observed in a case of hydrometra, recorded by Mr. J. M. Coley, of Bridgnorth, in the 4th Vol. of the Transactions of the Provincial Medical and Surgical Association.

With respect to ovarian tumors, the author remarks that those containing fluid may be diminished by puncture ; while those of a malignant nature could only be prolonged with accompanying misery by the artificial evacuation of the uterus. In proof of the failure of this operation an instance is brought forward, in which the induction of premature labour at the fifth month was followed by death the next day. It does not appear however that the fatal result was altogether, if at all, attributable to this cause; as the life of the patient had been previously placed in imminent danger by an accident, and the operation was adopted as a last resource for the relief of an alarming peritonitis, accompanying a malignant ovarian tumor.

For the relief of ascites connected with pregnancy, every judicious practitioner must coincide with the author in preferring abdominal paracentesis to the artificial discharge of the uterine contents.

On the whole, this short Essay is highly creditable to the sound discretion and humanity of the author, and his conscientious feelings on so grave a subject cannot be more forcibly illustrated than by his own words in the concluding paragraph.

> " Under any circumstances, it will be most important to consider whether the disease is likely to prove fatal; for in sanctioning the operation we must not only believe that such a result will happen, if gestation is suffered to continue, but ought to entertain a strong conviction that the evacuation of the uterus is essential to the removal or the marked mitigation of the disease. As a matter of conscience, the concurrent testimony of several eminent midwifery practitioners in its justification should previously be obtained." 172.

5. Laceration of the uterus, according to the experience of some of the most distinguished practitioners in midwifery, as Hunter, Denman, Ramsbotham, &c., was considered uniformly and necessarily fatal. As however several successful cases have of late been recorded by professional gentlemen of unquestionable veracity, we ought never to despair of recovery, until life has actually become extinct.

It has been long since ascertained that those most liable to this accident are women who have given birth to several children, and an attempt has been made to explain this circumstance, by supposing that after repeated pregnancies the parietes of the uterus become attenuated, especially in cases of distorted pelvis. This appears to be the opinion of Dr. John Ramsbotham from the following passage;

> " I am thence led to suspect, either that the uterus has received some local mechanical injury from the violence of its own efforts or from the previous effects of artificial assistance, by which its structure is, at this point weakened ; or, that it is *thinned at the part where it gives way during the last months of gestation, by continued pressure against some prominent part of the pelvis.*"—*Practical Observations on Midwifery.* Part 1, page 383.

It is the opinion of Mr. Ingleby that this misfortune is more likely to arise from the projection of the sacrum observable in a distorted pelvis during pregnancy, than from this supposed attenuation of the uterus.

"In the disease termed *malacostion*, the sacral promontory undergoes a sensible change in its figure, *antecedent* to any apparent softening of the pelvic bones in general, and the reason is obvious. This yielding, which is at first very slight and is arrested after delivery, returns with a recurrence of pregnancy; rendering each act of parturition more difficult."

Thus we understand why lacerations occur more frequently in women who have borne children, than in primiparæ; and the explanation is far more consistent with the principles of physiology, than to ascribe the injury to attenuation of the uterine tissues. "In confirmation of this," says Mr. I., "I have the satisfaction of adducing the experience of my valued and intelligent friend, Mr. J. M. Coley of Bridgnorth. In his description of two cases, which he very kindly sent me, I find the following observation;"—

"'In both cases, the parietes of the uterus were unusually thick, a circumstance which leads me to doubt the truth of the opinion entertained by accoucheurs of the first rank, that this accident *generally* arises from an attenuation of the womb, occasioned by repeated pregnancies and the pressure of the projecting bones of the pelvis. In both instances, besides the remarkable firmness and thickness of the organ, the situation of the rupture would negative such an explanation of the cause of the injury. The contractions of the uterus were excessive, and in one of the cases the ergot was given in very large doses, and the forceps applied during strong pains.'" 175.

According to the author's experience, this accident is liable to be induced by the resistance of a scirrhus of the os uteri; and it has been known to arise from convulsions, and from exostosis of the ossa pubis.

Notwithstanding the preceding remarks on attenuation of the uterus, the author admits that rupture has occurred from this cause, as well as from softening of that organ and ulceration of its lining membrane.* The laceration of the peritoneal coat he attributes to the irregular contraction of the uterine fibres.

After alluding to the different situations in which laceration of the uterus and vagina have usually been met with, the author proceeds to notice the circumstances indicating the probability of the accident occurring, and the steps necessary to prevent it. For information on this head, we must refer our readers to the work itself, where they will find useful remarks, the result of extensive experience.

The symptoms of lacerated uterus and vagina are next detailed; but, as these are familiar to the profession, we pass on to the treatment of these unfortunate cases. We cannot, however, leave unnoticed an extraordinary case, which occurred in the practice of Mr. Cook, of Coventry, as we have seen the exfoliated uterus alluded to, and can vouch for the accuracy of the statement.

"The absolute separation of the whole uterus from the vagina, as the result of laceration or sloughing, would seem quite incompatible with the preservation

---

"In one instance, Dr. Collins found the accident to arise from a venereal ulceration about the cervix uteri."—*Treatise on Midwifery*, p. 287.

of life; but an instance of this kind, which terminated favorably, has been recorded by Mr. Cook, of Coventry. The separation took place the second day after delivery, and the specimen, which embraces the uterus in a state of inversion, together with its ligaments and a small portion of the vagina, has just been deposited in the anatomical museum in this place. The perfect recovery of the patient constitutes the singular and interesting feature of the case." 195.

"In treating uterine laceration before delivery, and in the last three months of gestation, we have to consider, first, the condition of the uterine orifice; secondly, the situation of the child; and, thirdly, the state of the general system. Respecting the treatment of this injury, under its least complicated form, by immediate delivery, little need be said. It is generally acknowledged in this country, that if the head be accessible to the forceps, it should be promptly abstracted—and if above the brim, provided it does not retreat when moderate pressure is applied against it, or can be sufficiently steadied by pressing the uterus externally, perforation must always supersede the dangerous operation of version in the abdomen." 197.

Immediate delivery, by introducing the hand through the fissure, is usually practised. In one instance, where this course was adopted, the author states that delivery was followed by a large protrusion of the intestines, and the speedy death of the patient. Gastrotomy will occasionally be found the best mode of proceeding. When the os uteri is not fully dilated, and the state of the patient will admit it, the author advocates the propriety of effecting dilatation without delay. With respect to gastrotomy, where such an expedient is indicated, it must be obvious that less risk will be incurred by it, than by allowing the child to remain within the abdominal walls; and several cases have been reported, in which the life of the mother has been thus preserved. It should not be undertaken while the patient is in a state of collapse, threatening instant destruction; but, at the moment the accident has happened, or after such a degree of re-action has taken place as may safely admit of manual operation. The life of the child is, in our opinion, of secondary importance in these cases; therefore it should be our main object to seize the critical moment most favourable for the safety of the mother, both on her own account, and that of the operator's reputation.

When the diameter of the pelvis is so reduced as to require the aid of craniotomy, it is questionable whether an opening, made into the abdomen, would not be safer than the tedious and injurious means often adopted to accomplish extraction by the former proceeding.

To promote the contraction of the lacerated parts, when recovery appears probable, the author advises the application of a healthy infant to the breasts of the patient, and to the abdominal surface a suitable compress.

Isolated, and surrounded by an envelope of varying consistency, acquired during its extra-uterine residence, the fœtus has been found incarcerated in the abdomen of the mother during many years, without occasioning any injury to her health, or shortening the duration of her after-life. An instance of this kind has been under the notice of our friend, Mr. J. M. Coley, of Bridgnorth, during a period of more than 30 years; and, as the patient has requested him to explore the contents of the abdomen after death, should he survive her, he will have an opportunity of recording the appearance of this ancient cyst and its contents.

As the after-treatment must necessarily be conducted on the general principles of preventing and subduing inflammation, whether the child may have been extracted or not, we need not follow the author in his detail of these particulars.

Nine cases of ruptured uterus and vagina are adduced by the author, seven of which were fatal, and two terminated in recovery. One of the favorable cases of rupture of the uterus occurred in the practice of Mr. Evans, who was assisted by Mr. Taylor and Mr. Ingleby in effecting delivery by craniotomy. The other happened to a patient of Mr. J. M. Coley's, at Bridgnorth. The laceration in this instance extended from the cervix nearly to the fundus, and the whole of the fœtus, except the feet, had escaped from the uterus. Mr. Coley, seizing the feet without loss of time, had no difficulty in effecting delivery.

6. Inversion of the uterus may be traced to the rude separation of the placenta, sudden expulsion of its contents, when the funis is unusually short, or the exit of a polypus during delivery, or when the uterus is in an unimpregnated condition. The author has also heard of two cases, in which inversion took place spontaneously immediately after the birth of the child.

After describing the usual kind of inversion, the author states that he has met with a variety occurring in the course of the longitudinal fibres, which, he believes, has not been noticed by other writers. The following he refers to as an instance.

"I was compelled, in conjunction with another practitioner, to apply the forceps under the disadvantage of uterine inertia. After the delivery of the child, there was no tendency to expel the placenta; but a portion of the mass having separated, a slight effort was made with the funis. The placenta descended considerably beyond the os internum, together with a quantity of the uterus, apparently the whole length of its right side, the left not being sensibly depressed. Flooding ensued. At the moment we were rather perplexed; but the nature of the displacement became evident, and the inverted part was immediately returned together with the placenta. The adherent portion of the mass was then separated without delay, and the case treated in the usual manner." 223.

The plan laid down by Merriman for the treatment of inversion, with the placenta adhering, appears to the author the best which can be followed, *i.e.* to reverse the uterus with the placenta, or, if impracticable, to pull off the latter, and replace the uterus without delay. The mode of returning the uterus is next described with much care and judgment. Should this organ be found inflamed, the author, who has only seen one instance of this kind, recommends delay, and the application of leeches and other appropriate applications.

Unless inversion is speedily noticed and replaced, the contraction of the os uteri renders it afterwards a difficult or impossible proceeding. Hence these cases have been too frequently abandoned to their fate. Several instances have, however, occurred, in which, at different periods after delivery, practitioners have succeeded in replacing the organ within its proper bounds. In one case, success followed the attempt at the end of twenty-four hours; in another, at the end of two days; and, in another, at the end of twelve weeks. Encouraged by these facts, our ingenious and persevering author also succeeded in returning an inverted uterus at the end of a week after the accident occurred, and while the patient was in a state of collapse, and apparently moribund.

The inverted uterus in one instance, noticed in a former part of this article, has been known to slough away, the patient recovering without any farther injury.

When inversion of the uterus is of long standing, and its replacement is found impracticable, the patient is in constant misery, especially in the erect posture, or when the body is bent forward in any laborious occupation. Hence, the entire removal of the inverted part by the knife or ligature has been proposed and practised; and several successful operations of this nature are on record. The only danger apprehended from the knife by the author is hemorrhage, but he thinks that may be commanded by plugging the vagina with lint and oil, and by cold applied over the pubes. The patient may obtain relief by several contrivances; but the most effectual remedy is the old-fashioned pessary, described, we think, by Wiseman.

By means of this instrument, Mr. Coley, of Bridgnorth, to whom we have before alluded, succeeded in affording effectual relief to a patient, who had laboured under complete inversion during seven years, occasioned by a rude and forcible extraction of the placenta. The whole of the uterus was inverted through the os uteri, and hung between her thighs, and the fundus was ulcerated to the extent of a half-crown piece, and discharged an offensive matter. The inverted uterus and the os uteri, forced through the os externum, were exposed to view, and presented the appearance of a large malignant tumor, of the size of a cocoa-nut. By applying a gradual and gentle pressure, he succeeded in replaçing the inversion, and the descent of the prolapsed os uteri was prevented by the pessary in question, which was secured round the hips by means of tapes. The patient was thus rendered quite comfortable, and the ulcer healed in a short time. She could now walk any distance without inconvenience, and the formidable operation of excision, which Mr. Coley had proposed, and to which she had consented, was thus fortunately superseded.

7. In the essay on the signs of pregnancy, the author's object is not to enter at large on the symptoms in general, but to animadvert briefly on the principal evidences, real or supposed, of the pregnant state; on the difficulty of forming a diagnosis; and on the circumstances denoting the cessation of life in the infant.

A. Vomiting usually ceases after quickening, and always after the death of the ovum. The cessation of sickness in the latter case may be explained, on the supposition that the stomach recovers its customary supply of blood and nervous energy, of which the process of fœtal germination had deprived it. Vomiting is generally more severe in first than subsequent gestations.

B. Uterine hæmorrhage, especially in the early months of pregnancy, is commonly destructive of the life of the embryo; yet instances have repeatedly occurred, of a sanguineous discharge continuing or returning periodically nearly through the whole period of utero-gestation.

C. In some women, but certainly not in all, soon after conception has taken place, the breasts become hard, tender, and painful; and we have observed this condition of the breasts accompany the development of an imperfect, as well as a perfect ovum. The dark areola commonly occurring, the author states, is the principal characteristic, and is most distinguishable in persons of a dark complexion. A scaly state of the areola, and a mottled appearance of the breast, are also considered by him as marks of pregnancy. The secretion of milk is an equivocal symptom.

D. In twin cases, one of the fœtuses may die at any early period of pregnancy, and yet be retained in the uterus until the expiration of the natural term of utero-gestation. When premature expulsion of a twin occurs, the uterus expels the second fœtus in the course of a few hours or days. This is dependent on a principle inherent in that organ, noticed in our review of the author's work on hæmorrhage, which ensures a continuation of the expulsive efforts after a portion of its contents has been evacuated; and it applies to the discharge of hydatids or other imperfect ova, as well as the most perfect production.

E. Fetor of the discharges may precede or accompany the birth of a living or dead child, and is, therefore, no criterion of the loss of vitality.

F. The ascent of the uterus and the state of the os and cervix uteri, next attract the author's attention; but, as we meet with nothing of importance to detain us, we pass on to—

G. The state of the hypogastrium and the examination by the vagina. Having described the mode of exploring the hypogastric region, the author proceeds to the vaginal investigation. The most convenient position of the patient may be found to be the supine or lateral one; and, as he very correctly observes, the upright posture will occasionally be required to afford us positive information. At the end of the fifth month the uterine tumor is on examination distinctly perceptible in the hypogastric region.

Of all the means we possess of ascertaining the existence of pregnancy from the third to the fifth month, which is the usual period, when our diagnosis is solicited, the examination by the vagina is the most to be relied upon, if conducted by an experienced practitioner. Here our author, after describing the resiliency which the gravid uterus may be made to present, has omitted to notice other unequivocal proofs of pregnancy, *i. e.* the fluctuation and solidity, which a tutored finger may discover between the cervix uteri and the pubes, and the increase of diameter, which the upper part of the vagina will be found to have acquired.

Among the various diseases resembling pregnancy the author mentions enlargement of the spleen and liver, a polypous tumor, tuberculated peritoneum, fatty depositions, tympanitis of the uterus, ovarian dropsy, and fibrous tumors. Polypous tumors sometimes acquire a considerable magnitude. The author possesses one, which he says cannot weigh less than four pounds. The presence of such a disease, whether adherent on all its surface to the uterus or not, may be readily distinguished from fœtal impregnation by its solidity and other symptoms. When impregnation takes place, while a polypus lies concealed in the uterus, the growth of the fœtus and polypus will proceed at the same time.

H. Obliquity of the uterus may proceed from deformity of the pelvis and spine, distention of the colon, relaxation of the abdomen and retention of urine.

When the oblique position of the uterus renders pregnancy doubtful and hæmorrhage supervenes, the author inquires whether we should be justified in dilating the cervix uteri for the purpose of ascertaining the existence of gestation as early as the third or fourth month, and of rupturing the membranes. He is of opinion that we are, on account of the occasional fatality resulting from uterine hemorrhage at these early periods of utero-gestation. "Whether, then, the signs of pregnancy be conclusive, or only strongly presumptive, the propriety of passing the hand into the vagina and dilating the uterus, with the view of ascertaining its contents (provided hæmorrhage is pro„ tracted and dangerous to life,) must, I think, be admitted as a rule of practice. 260.

I. The abdominal tumefaction may, after a certain period of pregnancy, continue stationary through the remaining months. An instance of this kind is related by the author, which arose from the early rupture of the membranes and the continued discharge of liquor amnii. It appears at other times to proceed from a defective secretion of liquor amnii.

K. The combination of pregnancy with ovarian disease is not an unfrequent occurrence; and when hydrops ovarii accompanies gestation it sometimes happens that paracentesis of the abdomen becomes necessary. When the ovarian cyst extends to the vagina, this operation may be required to prevent the accidental rupture of the cyst during parturition. In one case the author punctured the sac through the vagina and extracted four gallons of gelatinous fluid.

Ascites is rarely associated with pregnancy. When this combination does exist, it may be requisite to relieve the former by puncture during the pregnant state, and several cases have occurred, in which this operation has been performed with perfect safety.

A communication between an ovarian cyst and the vagina is sometimes met with, and the author believes that a thin colourless fluid is occasionally discharged from the lining membrane of the uterus during and after pregnancy.

The subject of hydrops amnii, or dropsy of the amnion, is next slightly alluded to. This disease has attracted the notice of several authors, particularly Mauriceau, Lamotte, Baudelocque and Gardien. Mercier attributed the inordinate secretion to an inflammation of the amnion; while others, who have carefully examined this membrane, declare that no marks of inflammation could be traced in it. We believe it is the general opinion of the best modern pathologists, that it is a disease of the ovum, unconnected with the condition of the uterus. The diagnosis of this affection can only be arrived at with certainty by an examination per vaginam. When the finger is applied to the space between the os uteri, and the pubes, and percussion of the abdomen is effected, an immediate and distinct fluctuation will be perceived in the former situation. In this case also the cervix uteri will be found almost completely obliterated by the great expansion of the whole uterus.

L. The abdomen now and then undergoes a remarkable diminution during the pregnant condition of the uterus. This may proceed from the death of the fœtus, which notwithstanding may be retained from the third month to the full period of utero-gestation. It may also be explained by the rupture or expulsion of an adventitious sac from the uterus during gestation. May not these pouches consist of imperfect ovarian vesicles, which may have descended through the fallopian tube with the impregnated ovum? The subsidence of the abdomen may be dependent on the escape of part of the liquor amnii through an aperture at the side of the membrane, which, when occurring spontaneously, we know will frequently not interfere with the progress of gestation. The discharge of fluid from the chorion will be too insignificant to deserve our notice.

M. Irregularity in the form of the abdomen may, according to our author, be produced by an irregular development of the uterus or fœtus, with a deficiency of the amniotic fluid. He is also inclined to suspect that it may arise from a separation of the recti muscles, or other faulty state of the abdominal parietes.

N. The motion of the fœtus, which is sufficiently characteristic without any description here, may be considered as the only positive evidence of fœtal existence. Yet the crawling movement has now and then been confounded with the action of the abdominal muscles, and the peristaltic elevations of the intestines. The experienced author, in his usual candid manner, confesses that he was himself deceived on one occasion by the latter circumstance, in a case which Mr. J. M. Coley, of Bridgnorth, had previously pronounced to be one of a malignant character and unconnected with pregnancy.

"I was desired to see a woman, in whom a tumour had developed itself on the left of the umbilicus, and on a line with it. It was slightly moveable, at times painful, but not on pressure; projecting considerably forward, sloping from its summit, and being about the size of the gravid uterus at the fifth month of pregnancy. Its texture appeared to be firmer in some parts than others, but this was owing to partial adhesions having formed between the general and uterine peritoneum. It afforded no sense of fluctuation, but on placing the hand over the surface of the tumour, a distinct crawling movement was traced in every part of it, but unaccompanied by the sudden impulse" (the ictus) "before alluded to. On internal examination, although the os uteri and the vaginal portion of the cervix were scarcely changed, the superior part of the cervix had degenerated into a tumour, quite as large as a child's head, exceeding it in firmness, but without the resilient property of an immature fœtal skull. Repercussion could not be produced, and the stethoscope afforded no evidence whatever of pregnancy. The tumour continued enlarging—the sufferings of the patient progressively increased, and the result was fatal. On examination, *post mortem*, it was found that the inferior part of the uterus had degenerated into a thick and fibrous, or fibro-cartilaginous substance. The fundus and body of the organ were converted into a large sac, not thicker than an ox's bladder, and contained three pints of dark, fœtid, muco-purulent fluid, the consistence of gruel. The mucous and fibrous structures of these parts of the uterus had nearly disappeared, and the peritonæal coat, though generally thickened, had become very thin in one spot, where it burst during the examination, and allowed a part of the fluid to pass into the abdomen. The fluid was prevented during life from escaping into the vagina by a quantity of tenacious mucous" (mucus) "which lined the sides of the cervix uteri, as in ordinary pregnancy." 277.

Hydatids have also been found to convey a sensation to the hand laid over the abdomen, resembling that of a living fœtus.

The colon is liable in females of hysterical habit to be thrown into a convulsive state from an irregular peristaltic motion of its muscular fibres, which, from the spherical form and firmness of the tumefaction, might be mistaken for an uterine tumor or pregnancy. Cases of this kind have been observed, particularly by Morgagni and Valsalva, in which an irregular motion, resembling the ictus of a fœtus or the pulsation of an aneurism, has been present. We have frequently met with these occurrences, and must confess, at first, were as much astonished as our author at the sudden manner in which these firm and globular swellings have taken place.

The movements of the fœtus have, the author states, been suspended by hyoscyamus during nine or ten hours, and almost paralyzed by the full and continued action of ergot of rye.

Having made some remarks on the ordinary signs of death in the fœtus, the author next introduces the subject of—

O. Auscultation. By this valuable auxiliary the vitality of the fœtus may be ascertained at any period previously to birth from the commencement of the fifth month. The author believes the *soufflet* an uncertain evidence of pregnancy. The fœtal pulsation, which varies from 100 to 170, diminishes in frequency after the liquor amnii has been discharged. The action of the fœtal heart is sometimes too feeble to be distinctly heard. This was the case in a fœtus, before the Cæsarian operation was performed, in which instance the extraction of a living child confirmed the correctness of the mother's sensations. The diminution of the heart's impulse may, according to our author, be occasioned by the strong contraction of the uterus during severe pain, and disappear on the pain subsiding.

P. On the state of the funis during labour the author has nothing to add to our present knowledge on the subject.

Q. Various circumstances have a tendency to produce the death of the fœtus in utero; as a partial separation of the placenta, maternal fear, long-continued asphyxy, malignant fevers, external violence, &c. The most frequent of all the causes is certainly the venereal poison, which may remain for years or through life in a latent form, and yet be sufficiently active, sooner or later, to extinguish the spark of uterine existence in a succession of contaminated embryons. We have known this poisonous influence manifested on sixteen human germs in succession, the parents remaining apparently healthy, and, as it were, restoring their venomous constitutions at the expence of their offspring. The usual course of events is this: the husband contracts disease one, two, or more years before marriage, and, to all external appearance, is cured long before the marriage-ceremony is performed. Feeling conscientious on the subject, he takes the opinion of a surgeon, who assures him that, being free from all apparent infection, he may enter upon marriage with perfect security. Should his wife become pregnant, the probability is, that the fœtus will be expelled in a rotten state about the sixth or seventh month. Should conception not take place, it will be found that her system becomes poisoned, and syphilitic symptoms supervene of various character. The husband continues to enjoy his accustomed health: the virus appearing in him to have been modified by medicine, and rendered innocuous to his own frame. On this subject the author appears to us to entertain very sound views, and recommends both parties to undergo a mercurial treatment. The malignant influence of latent disease, transmitted from either parent to the fœtus, attracted the notice of the late Mr. Hey of Leeds, who published an essay on the subject, we believe, in one of the early volumes of the Medico-chirurgical Transactions. The period at which the elements of a disease, destined to annihilate the vitality of the human germ approaching to fœtal maturity, are absorbed from the maternal circulation, is uncertain. The author is of opinion that the virus is innocuous to the embryo during the first few weeks of his existence, and

"That when the child has acquired a size common to the sixth or seventh month, the changes wrought upon it through the placenta are unequal to maintain its vitality unimpaired." 293.

Mr. Ingleby's sentiments on the propriety of prescribing mercury to the mother in these cases, while in a state of pregnancy, correspond with our experience, which has been uniformly successful. When the mercurial treatment is carefully conducted, we have no hesitation in recommending its adoption at any stage of utero-gestation; and have invariably found this proceeding not only conduce to the safety of parent and child, but obviate the premature expulsion of the uterine product.

Having now completed our review of Mr. Ingleby's interesting book, it remains for us to add a few concluding remarks. The work before us is not one of those elementary compilations, which we meet with every day, adapted only for the initiation of the medical student: it is a production, which may be read with satisfaction and advantage by the most experienced practitioner and teacher; being the result of extensive occupation in the obstetrical department, assisted by reading and sober reflection. The liberal spirit which pervades the volume is deserving of imitation in medical writings; and, although we have ventured to differ in opinion with the author on a few points, we can recommend his publication as a safe and useful guide in practice.

